# The impact of glaucoma on quality of life in Ethiopia: a case-control study

**DOI:** 10.1186/s12886-017-0643-8

**Published:** 2017-12-13

**Authors:** Fisseha A. Ayele, Banchamelak Zeraye, Yared Assefa, Kbrom Legesse, Telake Azale, Matthew J. Burton

**Affiliations:** 1 Department of Ophthalmology, School of Medicine, College of Medicine and Health Sciences, University of Gondar, P O Box 196, Gondar, Ethiopia; 2 Department of optometry, School of Medicine, College of Medicine and Health Sciences, University of Gondar, Gondar, Ethiopia; 3 Department of Health Education and Behavioral Sciences, Institute of public health, College of Medicine and Health Sciences, University of Gondar, Gondar, Ethiopia; 4 International Centre for Eye Health, Faculty of Infectious and Tropical Diseases, London School of Hygiene & Tropical Medicine, London, UK

**Keywords:** Glaucoma, Quality of life, GQOL-15, Gondar, Ethiopia

## Abstract

**Background:**

Glaucoma is a chronic disease characterized by irreversible optic nerve damage and visual field loss that leads to visual impairment and blindness; ultimately limiting personal independence and compromising overall quality of life of affected individuals. There is paucity of information on how glaucoma affects the quality of life of patients in low and middle-income countries where resources for both diagnosis and treatment of such conditions are limited. In this study we investigate the impact of glaucoma on quality of life in Ethiopian patients.

**Methods:**

The quality of life of 307 glaucoma patients and 76 normal controls that were frequency matched to the age and sex profiles of the cases was assessed using Amharic version of Glaucoma Quality of Life −15 questionnaire. Linear regression models and the t-test were employed to compare significant differences in GQL-15 scores and to generate mean and mean differences between cases and controls respectively.

**Results:**

The mean GQL-15 score in the glaucoma cases was substantially higher (indicating poorer quality of life) than the controls [cases 46.3 (95% CI, 28.8–63.8) and controls 18.6 (95% CI, 15.2–22.0), *p* < 0.0001]. Cases with normal visual acuity and mild glaucoma had significantly higher scores than the controls. Poorer quality of life was associated with age ≥ 71 years old 51.1 (95%CI, 26.2–75.9), rural residence 55.7 (95%CI, 49.9–61.5), monthly income of <400 Birr (53.1; 95%CI, 50.5–55.6), diagnosis time 1–5 years (49.6; 95%CI, 41.2–57.9), severe visual impairment (70.5; 95%CI, 58.1–82.8), and advanced glaucoma (50.9; 95%CI, 43.6–58.3).

**Conclusion:**

These glaucoma patients, including those with normal visual acuity and early disease, had poorer quality of life compared to normal controls. Older age, rural residence, low income and more advanced disease were significantly associated with poorer quality of life. There is a need to increase awareness of the impact of glaucoma among clinicians, patients and their families, for a better understanding of the impact this disease has on a person’s life.

## Background

The World Health Organization (WHO) defines Quality of Life (QOL) as an “*individuals’ perceptions of their position in life in the context of the culture and value systems in which they live and in relation to their goals, expectations, standards and concerns*” [1]. QOL is a broad and complex concept. It is affected by a person’s physical health, psychological state and level of independence, social relationships and environment. Vision Related Quality of Life (VRQOL) specifically focuses on a person’s satisfaction with their visual function and how their vision impacts on their daily life [2].

A group of eye diseases that can particularly compromise VRQOL are the glaucomas. The glaucomas are characterised by damage to the optic nerve with corresponding visual field loss. Sight loss due to glaucoma is irreversible. Typically, patients initially loose the mid-periphery of their visual field, while central vision tends to be involved later. The patients become aware of a functional defect when visual field loss impinges upon or involves central vision [3, 4]. Patients with advanced glaucoma may experience difficulty in recognizing faces, navigating, reading, watching television, noticing objects in their peripheral vision, and adapting to different levels of lighting. Moreover, they are also at increased risk of falls and accidents [5].

Besides the visual impairment (VI) and field loss, living with a chronic disease like glaucoma may negatively impact an individual’s QOL through greater dependence on other individuals for basic daily activities, stress/concern about the diagnosis, the need for lifelong treatment and follow-up, and the cost and side effects of treatment [6].

According to the WHO, glaucoma is the second leading cause of avoidable blindness after cataract, contributing 8% of total blindness worldwide [7]. This figure could be as high as 15% in some low- and middle-income countries (LMIC), particularly in Sub-Saharan Africa [8]. The Nigeria National Blindness and Visual Impairment Survey found that Glaucoma blindness was the second most prevalent blinding condition after cataract [9]. In the Ethiopian National Blindness and Low Vision Survey, which was conducted in 2005, glaucoma was found to be the fifth leading causes of blindness in Ethiopia (contributing 5.2% to the total blindness) [10]. The fact that the survey included individuals with visual acuity worse than 6/18 in either eyes and the exclusion of patients with corneal opacity from intra ocular measurement could have resulted in underestimation of the prevalence of glaucoma.

There is limited information on how the QOL of individuals is affected by glaucoma in LMIC like Ethiopia, where resources for both diagnosis and treatment are limited. In this case-control study we investigate the impact glaucoma has on the QOL of Ethiopian individuals with this disease.

## Methods

### Ethical considerations

This study was reviewed and approved by the Ethics Committee of the College of Medicine and Health Sciences, University of Gondar, Ethiopia. The nature of the study was explained to potential participants. Individuals were enrolled into the study if they gave informed verbal consent. No names or identifying information were recorded on the questionnaires. Interviews were conducted in private, and all subjects were assured of confidentiality.

### Study participants

Participants were recruited at the Ophthalmology Outpatient Department, University of Gondar Eye Care and Training Centre, Amhara Region, Ethiopia. In this study the cases were consecutive individuals with a diagnosis of glaucoma attending the department between April 24 and May 27, 2015. We excluded individuals with glaucoma who also had (i) high refractive errors (greater than ±5 Diopters), (ii) visually significant cataracts (greater than Stage 2 LOCS III classification) [11], (iii) any other visually significant ocular pathology, (iv) a history of intraocular surgery (incisional or laser) in the preceding 3 months. We also excluded individuals with a major systemic illness or physical disabilities that could affect response to GQoL-15 questionnaire.

The control participants were also recruited from patients attending the same Ophthalmology Outpatient Department. The controls had a visual acuity of 6/9 or better (+/− correction) and otherwise healthy eyes. Pseuodophakic individuals with a visual acuity of 6/9 or better were eligible. We excluded individuals who had (i) high refractive errors (greater than ±5 Diopters), (ii) visually significant lens opacities (greater than Stage 2 LOCS III classification), (iii) suspicious optic discs, or (iv) corrected visual acuity of less than 6/9. We also excluded individuals with a major systemic illness or physical disabilities that could affect response to GQoL-15 questionnaire. The controls were recruited after the case, between February 1 and May 31, 2016. We recruited one control for every four cases; they were frequency matched to the age and sex profile of the cases.

### Clinical assessment

Cases and controls underwent a standard ophthalmic examination as part of their routine clinical care at the department. The presenting distance visual acuity (VA), with spectacle correction if available, was measured for each eye separately using a tumbling-E Snellen visual acuity chart at six metres. Pinhole test was done for those with visual acuity 6/18 or worse. If participants were unable to see this at six metres, the testing distance was reduced to three metres. If they failed to see any letters at three metres, vision was assessed for “counting fingers” (CF), “Hand Movement” (HM) or “Perception of Light” (POL). Presenting distance visual acuity was defined by the revised WHO classification,; patients were categorized according to the vision in the better seeing eye [12]:

- *Mild or no visual impairment*: presenting distance visual acuity equal to or better than 6/18
- *Moderate visual impairment*: presenting distance visual acuity worse than 6/18 but equal to or better than 6/60
- *Severe visual impairment*: presenting distance visual acuity worse than 6/60 but equal to or better than 3/60
- *Blindness*: presenting distance visual acuity worse than 3/60

The eyes were examined by an ophthalmologist specialised in glaucoma (2 years fellowship training in glaucoma). The anterior segment was assessed using a slitlamp bio-microscope, intraocular pressure (IOP) measured by Goldman tonometry, the anterior chamber angle was examined by gonioscopy and stereoscopic optic nerve head evaluation performed using a 90D (Volk Optical Inc.) indirect lens.

Glaucomatous Optic Neuropathy (GON) was defined based on the scheme developed by the International Society of Geographical and Epidemiological Ophthalmology [13]. Cases were diagnosed if one or more of the following optic nerve head changes were observed in either eye: (1) vertical cup to disc ratio (VCDR) of ≥0.7 or (2) a difference in the VCDR of ≥0.2 between the 2 eyes or (3) the narrowest remaining vertical neuro-retinal rim of ≤0.1 disc diameter. We did not include visual field (VF) assessment in the diagnosis and staging of glaucoma in this study as the test was only performed by a minority of our patients.

The following operational definitions for the sub-types of glaucoma were used [13]**:**

- *Primary open angle glaucoma (POAG)*: optic nerve damage meeting any of the three categories of evidence above, in an eye which does not have evidence of angle closure on gonioscopy, and where there is no identifiable secondary cause.
- Pseudoexfoliative glaucoma (XFG) is open angle glaucoma associated with characteristic pseudoexfoliative material deposited at the pupil margin or anterior surface of the lens; on gonioscopy there may be with hyperpigmention of the trabecular meshwork or exfoliative material in the angle.
- Chronic angle closure glaucoma (CACG) is optic nerve damage meeting any of the three categories of evidence above, in an eye which have evidence of angle closure on gonioscopy, and where there is no identifiable secondary cause.

The severity of glaucoma was staged using the Canadian Ophthalmological Society evidence-based clinical practice guidelines for the management of glaucoma in the adult eye [14]:

- Early Glaucoma: Early glaucomatous disc features (e.g., VCDR 0.7) and (or) mild VF defect not within 10° of fixation (e.g., MD better than −6 dB on HVF 24–2)
- Moderate Glaucoma: Moderate glaucomatous disc features (e.g., VCDR 0.75–0.85) and (or) moderate VF defect not within 10° of fixation (e.g., MD from −6 .to −12 dB on HVF 24–2)
- Advanced Glaucoma: Advanced glaucomatous disc features (e.g. VCDR >0.9) and (or) VF defect within 10° of fixation (e.g., MD worse than −12 dB on HVF 24–2)

### Structured interview

A structured, pre-tested questionnaire that consisted of questions on socio-demographic characteristics, clinical assessment of glaucoma and the Glaucoma Quality of Life-15 (GQL-15) questionnaire was used to collect data [15]. The questionnaire was translated into the local language (Amharic) and then back translated into English by expertise and 5% of the questionnaire was pre-tested. Data were collected through face to face interviews (most study participants were illiterate) and medical chart review.

The GQL-15 is a user friendly 15-item questionnaire made up of four domains of perceived visual disability: (1) central/near vision, (2) outdoor mobility, (3) peripheral vision and (4) glare / dark adaptation [16]. There are different numbers of question items in the four domains: central/near vision (2 items), outdoor mobility (1 item), peripheral vision (6 items) and glare / dark adaptation (6 items). Therefore the maximum potential score the domains also vary. It is a well validated tool with high internal consistency and test-retest reliability [15, 17]. The GQL-15 has a scoring scale ranging from 1 to 5, where 1 stands for “no difficulty in performing the activity” and 5 for “severe difficulty due to visual reasons”. If patients do not perform a specific activity for reasons other than impaired vision, a score of 0 is given for that task. This can yield a total score of between 0 and 75. Poorer GQL-15 and increasing difficulty with vision-related activities were associated with higher subscale scores [17–20].

### Data analysis

The coded data were checked, cleaned and entered into Epi Info version 7 and then exported into SPSS Version 20 (SPSS, Chicago III) for analysis. Frequencies, percentage, mean and standard deviation were used to describe study results as required. To calculate the subscale scores for the four domains of the GQL-15, the item level responses were scored on a numerical interval scale ranging from 0, indicating no difficulty, to 5, indicating severe difficulty. The subscale score for each domain was calculated using an average of the scores generated for the component item-level responses. Comparisons of cases and controls were done after adjusting variables for age and sex. Linear regression models and the t-test were employed to compare significant differences in GQL-15 scores and to generate mean and mean differences between cases and controls in each GQL-15 subscale and domain, respectively. Univariable analysis was first conducted for each variable. Variables that satisfied *p*-value <0.2 were selected for further analysis. The strength of association was interpreted using confidence interval. *P*-value <0.05 was accepted as statistically significant in this study.

## Results

### Socio-demographic characteristics

We recruited 307 glaucoma patients (cases) and 76 controls. The socio-demographic characteristics of the study participants are presented in Table 1. The age (*p* = 0.62) and sex (*p* = 0.87) distribution of the cases and controls were highly comparable. The mean age of the cases was 60.3 years (SD ± 14.0) and controls was 59.5 years (SD ±12.5). The large majority were married, with no evidence of a difference in marital status between groups (*p* = 0.06). Most were of Amhara ethnicity. Overall, the majority of participants lived in rural areas, however, there were proportionately more people from rural areas among the controls (75.0%) compared to the cases (55.4%) (*p* = 0.02). Educational level and occupation types were comparable between cases and controls. There was some evidence of a difference in income, with the controls earning slightly more than the cases (*p* = <0.0001).

**Table 1:** Socio-demographic characteristics of study participants

Variable	Cases		Controls		*p*-value*
	n/307	(%)	n/76	(%)	
Sex					0.87
Male	201	(65.5)	49	(64.5)	
Female	106	(34.5)	27	(35.5)	
Age					0.62
18–30	7	(2.3)	2	(2.6)	
31–40	22	(7.2)	5	(6.6)	
41–50	33	(10.7)	8	(10.5)	
51–60	61	(19.9)	15	(19.7)	
61–70	110	(35.8)	28	(36.8)	
≥ 71	74	(24.1)	18	(23.7)	
Marital status					0.06
Married	216	(70.4)	62	(81.6)	
Widowed	40	(13.0)	10	(13.2)	
Divorced	28	(9.1)	2	(2.6)	
Single	23	(7.5)	2	(2.6)	
Ethnicity					0.65
Amhara	298	(97.1)	73	(96.1)	
Tigre	9	(2.9)	3	(3.9)	
Religion					0.16
Orthodox Christian	283	(92.2)	64	(84.2)	
Muslim	19	(6.2)	12	(15.8)	
Protestant	5	(1.6)			
Residence					0.02
Urban	137	(44.6)	19	(25.0)	
Rural	170	(55.4)	57	(75.0)	
Education					0.20
None	138	(45.0)	42	(53.3)	
Informal (able to read and write)	93	(30.3)	18	(23.7)	
Primary school	29	(9.4)	7	(9.2)	
Secondary school and above	47	(15.3)	9	(11.8)	
Occupation					0.03
Government employee	56	(18.2)	15	(19.7)	
Farmer	130	(42.3)	43	(56.6)	
House wife	65	(21.2)	11	(14.5)	
Dependent on family	56	(18.2)	7	(9.2)	
Monthly Income (Ethiopian Birr)					<0.0001
< 400	119	(48.5)	10	(13.2)	
400–1000	110	(35.8)	42	(55.3)	
> 1000	48	(15.6)	24	(31.6)	

*p-values were calculated by t-test and adjusted for age and gender

### Clinical characteristics

The clinical characteristics of the cases are shown in Table 2. The majority had been diagnosed for between 1 and 5 years (194, 63.2%) and had bilateral disease (279, 90.9%). One hundred and thirteen (36.8%) had advanced glaucoma (determined by the extent of optic nerve damage) and 70 (22.8%) cases were either severely visual impaired or blind. The most frequent types of glaucoma were primary open angle glaucoma (164, 53.4%) and pseudoexfoliative glaucoma (111, 36.2%). Most of the control groups had a visual acuity of 6/6 (42, 55.3%) while the rest had 6/9. The main clinical diagnoses in the control groups include early cataract (24, 31.6%), corrected refractive errors (21, 27.6%) and pseudophakia with good outcome (16, 21.1%).

**Table 2:** Clinical characteristics of 307 cases with glaucoma

Variable	n/307	(%)
Duration of diagnosis		
< 1 year	75	(24.4)
1-5 Years	194	(63.2)
≥ 6 years	38	(12.4)
Duration of treatment		
< 1 year	156	(50.8)
1–5 years	130	(42.3)
≥ 6 years	21	(6.8)
Presence of other ocular disease		
Yes	149	(48.5)
No	158	(51.5)
Visual Acuity (better eye)		
Normal	111	(36.2)
Moderate visual impairment	126	(41.0)
Severe visual impairment	58	(18.9)
Blind	12	(3.9)
Type of glaucoma		
Primary open angle glaucoma	164	(53.4)
Pseudoexfoliation glaucoma	111	(36.2)
Chronic angle closure glaucoma	32	(10.4)
Stage of glaucoma		
Early	126	(41.0)
Moderate	68	(22.1)
Advanced	113	(36.8)
Laterality		
Unilateral	28	(9.1)
Bilateral	279	(90.9)

### Quality of life

The mean GQOL-15 scores of glaucoma cases and the controls are presented in Table 3. The mean GQL-15 score was substantially higher (indicating poorer quality of life) among the glaucoma cases than the controls (46.3 vs 18.6, *p* < 0.0001). The glaucoma cases had higher scores than the controls for all four domains (*p* < 0.0001).

**Table 3:** Distribution of the four subscales of the GQOL-15 scores in glaucoma cases and controls

Domain	Cases		Controls		Difference		*P* value*
	Mean	(95% CI)	Mean	(95% CI)	Mean	(95% CI)	
Central and Near vision	6.1	(3.5–8.7)	3.3	(2.5–4.1)	2.8	(2.5–3.1)	<0.0001
Outdoor mobility	3.2	(1.8–4.6)	1.3	(0.9 1.7)	1.9	(1.7–2.1)	<0.0001
Peripheral vision	19.3	(10.3–28.3)	7.2	(6.7–7.7)	12.1	(11.1–13.1)	<0.0001
Dark adaptation & Glare	17.7	(11.7–23.7)	6.9	(4.7–9.1)	10.8	(10.1–11.5)	<0.0001
Overall Score	46.3	(28.8–63.8)	18.6	(15.2–22.0)	27.7	(23.7–31.6)	<0.0001

*p-values were calculated by t-test and adjusted for age and gender

The relationship between GQL-15 scores and demographic characteristics among individuals with glaucoma are presented in Table 4. A higher total GQL-15 and domain scores (poorer QOL) were found for men, older people, illiterate individuals, rural residents, and people with lower monthly income. These same factors remained significant after adjustment in a multivariable linear regression model. The relationship between GQL-15 scores and clinical characteristics among the glaucoma cases are presented in Table 5. Increasing severity of glaucoma, longer duration of glaucoma and decreasing visual acuity were all associated with higher GQL-15 scores.

**Table 4:** Univariable and multivariable associations of glaucoma related quality of life with demographic characteristics

Variable	Domain									
	Central & near vision		Outdoor Mobility		Peripheral vision		Dark adaptation and glare		Overall score	
	Mean	(95% CI)	Mean	(95% CI)	Mean	(95% CI)	Mean	(95% CI)	Mean	(95% CI)
Age (years)										
18–30	3.7	(1.9–5.5)	1.9)	(0.9–2.9	11.6	(5.1–18.0)	12.4	(8.4–16.5)	29.6	(17.4–41.7)
31–40	2.9	(0.8–6.9)	1.8	(0.4–3.9)	11.5	(−2.2–25.5)	11.7	(3.0–20.4)	28.2	(2.1–54.3)
41–50	5.9	(2.2–9.7)	2.9	(0.8–5.0)	18.6	(5.0–32.1)	14.4	(6.0–23.0)	41.9	(16.3–67.4)
51–60	5.8	(2.1–9.4)	3.0	(0.9–5.0)	18.7	(5.4–32.0)	16.5	(8.2–24.9)	44	(18.9–68.9)
61–70	4.8	(2.9–10.2)	3.6	(1.6–5.6)	21.0	(7.9–34.1)	19.2	(11.1–27.5)	50.4	(25.7–75.0)
> 70	6.8	(3.2–10.5)	3.6	(1.6–5.7)	20.7	(7.5–33.9)	19.9	(11.7–28.3)	51.1	(26.2–75.9)
*P* value^a^	<0.0001		<0.0001		<0.0001		<0.0001		<0.0001	
*P* value^b^	<0.0001		<0.0001		<0.0001		<0.0001		<0.0001	
Sex										
Male	7.2	(6.1–8.4)	3.8	(3.2–4.5)	24.9	(20.8–29.1)	20.8	(18.1–23.6)	56.9	(48.8–64.9)
Female	6.1	(4.0–8.4)	2.8	(2.0–4.5)	19.7	(11.9–27.7)	17.9	(12.7–23.1)	47.1	(31.7–62.4)
*P* value^a^	0.02		0.03		0.02		0.03		0.01	
*P* value^b^	–		–		–		–		0.06	
Residence										
Rural	7.4	(6.5–8.2)	3.9	(3.4–4.3)	23.9	(20.9–26.9)	20.5	(18.5–22.5)	55.7	(49.9–61.5)
Urban	6.4	(4.9–7.8)	3.4	(2.5–4.1)	20.6	(15.2–25.5)	18.3	(14.8–21.7)	48.4	(38.3–58.5)
*P* value^a^	<0.0001		<0.0001		<0.0001		<0.0001		<0.0001	
*P* value^b^	<0.0001		<0.0001		<0.0001		<0.0001		<0.0001	
Educational status										
Unable to write and read	6.7	(6.3–7.1)	3.5	(3.3–3.7)	21.0	(19.6–22.3)	18.6	(17.6–19.5)	49.8	(47.2–52.4)
Only read and write	6.8	(5.8–7.7)	3.6)	(3.1–4.1	22.5	(19.0–25.9)	19.5	(17.0–21.8)	52.4	(45.7–59.0)
Primary school (1–8)	4.3	(3.0–5.6)	2.3	(1.6–3.0)	15.3	(10.6–19.8)	13.8	(10.6–17.0)	35.7	(26.9–44.5)
Secondary school (9–12)	4.8	(3.4–6.2)	2.1	(1.3–2.8)	13.5	(8.6–18.4)	17.3	(13.9–20.5)	37.7	(28.3–47.2)
Certificate and above	3.1	(1.7–4.5)	1.5	(0.7–2.5)	8.3	(3.4–13.1)	11.0	(7.0–14.3)	23.9	(14.6–33.2)
*P* value^a^	<0.0001		<0.0001		<0.0001		<0.0001		<0.0001	
*P* value^b^	<0.0001		<0.0001		<0.0001		<0.0001		<0.0001	
Monthly income (ETB)										
< 400	6.9	(6.6–7.3)	3.7	(3.5–3.9)	22.9	(21.6–24.2)	19.5	(18.6–20.4)	53.1	(50.5–55.6)
401–1000	5.6	(4.8–6.6)	3.1	(2.6–3.6)	18.3	(15.0–21.6)	16.7	(14.4–19.0)	43.8	(37.3–50.2)
> 1000	4.3	(3.3–5.5)	1.7	(1.1–2.3)	10.8	(16.8–14.6)	14.1	(11.4–16.9)	30.9	(23.2–38.6)
*P* value^a^	<0.0001		<0.0001		<0.0001		<0.0001		<0.0001	
*P* value^b^	<0.0001		<0.0001		<0.0001		<0.0001		<0.0001	

All *p*-values are calculated using linear regression

^a^ *P*-values from univariable linear regression analysis

^b^ *P*-values from multivariable linear regression analysis

**Table 5:** Univariable and multivariable associations of glaucoma related quality of life with clinical characteristics

Variable	Domain									
	Central and near vision		Outdoor Mobility		Peripheral vision		Dark adaptation and glare		Overall score	
	Mean	(95% CI)	Mean	(95% CI)	Mean	(95% CI)	Mean	(95% CI)	Mean	(95% CI)
Duration of diagnosis										
< 1 year	4.9	(4.3–5.4)	2.7	(2.3–3.0)	16.2	(14.2–18.2)	14.7	(13.4–16.0)	38.5	(34.6–42.3)
1–5 years	6.5	(5.2–7.7)	3.5	(2.7–4.1)	20.7	(16.3–25.1)	18.9	(16.1–21.7)	49.6	(41.2–57.9)
> 5 years	6.5	(4.9–8.0)	3.1	(2.1–3.9)	16.3	(12.9–23.8)	17.0	(13.5–20.6)	35.0	(34.4–55.4)
*P* value^ac^	<0.0001		<0.0001		<0.0001		<0.0001		<0.0001	
*P* value^b^	<0.0001		<0.0001		<0.0001		<0.0001		<0.0001	
Duration of treatment										
< 1 year	6.0	(5.6–6.4)	3.0	(2.8–3.2)	18.7	(17.2–20.1)	16.8	(15.9–17.7)	44.5	(41.8–47.3)
1–5 years	6.1	(5.1–7.1)	3.4	(2.9–3.9)	20.1	(16.5–23.6)	18.1	(16.5–21.1)	48.5	(41.6–55.3)
> 5 years	6.5	(5.0–8.1)	3.0	(2.1–3.8)	19.9	(14.3–25.3)	16.6	(13.1–20.3)	46.1	(35.3–56.8)
*P* value^ad^	<0.0001		<0.0001		<0.0001		<0.0001		<0.0001	
*P* value^b^	<0.0001		<0.0001		<0.0001		<0.0001		<0.0001	
Visual acuity										
Normal	4.8	(4.4–5.2)	2.5	(2.3–2.7)	15.2	(13.6–16.7)	14.8	(13.8–15.9)	37.3	(34.3–40.2)
Moderate VI	6.3	(5.3–7.3)	3.4	(2.9–4.0)	20.5	(17.0–24.3)	18.7	(16.1–21.2)	49.1	(42.1–56.0)
Severe VI	7.4	(6.3–8.6)	3.7	(3.0–4.3)	21.7	(17.6–25.9)	19.6	(16.8–22.5)	52.5	(44.5–60.4)
Blind	9.7	(7.9–11.4)	4.9	(3.8–5.9)	32.0	(25.5–38.4)	23.9	(19.6–28.3)	70.5	(58.1–82.8)
*P* value^a^	<0.0001		<0.0001		<0.0001		<0.0001		<0.0001	
*P* value^b^	<0.0001		<0.0001		<0.0001		<0.0001		<0.0001	
Glaucoma stage										
Early	5.3	(4.9–5.7)	2.8	(2.6–3.1)	16.9	(15.3–18.4)	16.2	(15.1–17.2)	41.2	(38.2–44.2)
Moderate	6.6	(5.4–7.8)	3.2	(2.6–3.9)	19.9	(15.7–24.0)	18.4	(15.6–21.2)	48.2	(40.1–56.2)
Advanced	6.7	(5.6–7.7)	3.5	(2.9–4.2)	21.8	(17.9–25.5)	19.0	(16.4–21.4)	50.9	(43.6–58.3)
*P* value^a^	<0.0001		<0.0001		<0.0001		<0.0001		<0.0001	
*P* value^b^	<0.0001		<0.0001		<0.0001		<0.0001		<0.0001	

*VI* visual impairment

^a^ *P*-values from univariable linear regression analysis

^b^ *P*-values from multivariable linear regression analysis

^c^ *P*-value results are for duration of diagnosis 1–5 years

^d^ *P*-value results are for duration of treatment 1–5 years

We performed a sub group analysis comparing cases with no or only mild VI to the controls [Table 6]. The mean GQL-15 scores for this sub-group of glaucoma cases still had worse QOL than the controls. Similarly, we compared the sub-group of cases with early glaucoma to the controls [Table 7]. This sub-group of cases had worse GQL-15 scores than the controls.

**Table 6:** Comparison of mean GQL-15 scores of glaucoma cases and controls with a presenting visual acuity of 6/18 or better in the better eye

Domain	Cases with normal visual acuity (*n* = 111)		Controls (*n* = 76)		Mean difference		*P* value^*^
	Mean	(95% CI)	Mean	(95% CI)	Mean	(95% CI)	
Central and Near vision domain	4.8	(4.4–5.2)	3.3	(2.5–4.1)	1.5	(0.9–2.1)	<0.0001
Outdoor mobility domain	2.5	(2.3–2.7)	1.3	(0.9–1.7)	1.2	(1.0–2.0)	<0.0001
Peripheral vision domain	15.2	(13.6–16.7)	7.2	(6.7–7.7)	8.3	(6.2–9.7)	<0.0001
Dark adaptation and Glare domain	14.8	(13.8–15.9)	6.9	(4.7–9.1)	7.9	(6.6–9.2)	<0.0001
Overall Score	37.3	(34.3–40.2)	18.6	(15.2–22.0)	18.7	(15.1–22.1)	<0.0001

*p-values were calculated by T-test and adjusted for age and gender

**Table 7:** Comparison of mean GQL-15 scores of early glaucoma cases and controls

Domain	Cases with Mild glaucoma (*n* = 126)		Controls (*n* = 76)		Mean difference		*P* value^*^
	Mean	(95% CI)	Mean	(95% CI)	Mean	(95% CI)	
Central and Near vision domain	5.3	(4.9–5.7)	3.3	(2.5–4.1)	2.0	(1.5–2.6)	<0.0001
Outdoor mobility domain	2.8	(2.6–3.1)	1.3	(0.9–1.7)	1.5)	(1.3–1.9	<0.0001
Peripheral vision domain	16.9	(15.3–18.4)	7.2	(6.7–7.7)	9.7	(7.7–11.6)	<0.0001
Dark adaptation and Glare domain	16.2	(15.1–17.2)	6.9	(4.7–9.1)	9.3	(7.8–10.7)	<0.0001
Overall Score	41.2	(38.2–44.2)	18.6	(15.2–22.0)	22.6	(18.7–26.4)	<0.0001

*p-values were calculated by T-test and adjusted for age and gender

## Discussion

In this study, using the GQL-15 assessment tool, we found that Ethiopian glaucoma patients had a poorer QOL compared to unaffected controls. Moreover, among the glaucoma cases, those with more advanced disease and poorer visual acuity had substantially poorer QOL scores. Onakoya et al. also demonstrated similar findings among Nigerian patients in their two separate reports [19, 20]. Multiple factors contributed to this glaucoma related reduced QOL. For example people with more advanced glaucoma will have peripheral and central visual impairment that could have an impact on their ability to move around, find objects, perform activities of daily living and adapting to changing lighting levels. They may be at increased risk of falls and accidents [5, 6].

The mean GQL-15 score difference between glaucoma cases and controls was significantly higher in domains of peripheral vision and dark adaptation and glare. This observation is consistent with earlier studies which have found that visual disability related to tasks associated with dark adaptation or glare, like walking after dark, seeing at night, adjusting to different levels of illumination and activities demanding peripheral vision such as avoiding tripping over and bumping into objects, seeing objects coming from the side and judging distances, are associated with impaired binocular visual field loss [15, 19–21].

A subgroup analysis found the mean GQL-15 scores of glaucoma patients with visual acuity of 6/18 or better and those with mild glaucoma were higher than the controls. This suggests that QOL can be reduced in glaucoma patients in this environment, even in the early stages of the disease. Similar observations have been made in other studies [15, 20, 22–24]. It is possible that factors other than the early functional visual loss, such as concern about the diagnosis and the need for lifelong treatment may also affect the perceived quality of, although the GQL-15 tool does not directly assess these issues.

We also observed that glaucoma patients who were rural residents with high illiteracy status to have poorer QoL. Generally, rural residents of developing countries like Ethiopia have poor access to eye care services [25]. Besides, the low literacy status might contribute to the poor health seeking behaviour observed in people living in rural areas that may result in the late diagnosis of the disease with severe visual impairment. Furthermore, most of the people from rural area had poor income to afford for their transportation and treatment related expenses.

We used the GQL-15 questionnaire, which is well validated with high internal consistency and test-retest reliability and had been used in Africa glaucoma patients. In one Nigerian study that compared the NEIVFQ25 (25-item National Eye Institute Visual Function Questionnaire) and the GQL-15 both appeared reliable in the assessment of QOL. They found the GQL-15 to be a shorter, simpler instrument, which could be easily administered in clinical practice [19]. The main limitation of this study was that it was cross-sectional; longitudinal study would have provided additional information about the changes in QOL throughout different stages of the disease.

Prevention of sight loss from glaucoma is particularly challenging in the African context. Patients frequently present late with advanced disease. Optometry services are not generally well developed and usually only available in larger urban centres. Therefore, there is relatively little opportunistic detection of glaucoma and simple, cost effective strategies are needed to find individuals with glaucoma before they develop substantial loss of vision. However, even when detected, further barriers to prevention of sight loss remain. Treatment is costly in relative terms and it is often difficult for the patient to sustain long-term access to medication. Therefore patient education and counselling are really important.

We found that glaucoma patients in Ethiopia, including those with normal visual acuity and early disease, have a poorer quality of life as compared to unaffected controls. Older age, rural residence, low income and worsening severity of the disease were significantly associated with poorer quality of life. This is important information to share with glaucoma patients. There is a need to increase awareness of the impact of glaucoma among clinicians, patients and their families, for a better understanding of the impact this disease has on a person’s life.

## Conclusions

It should be emphasized that the main goal in the management of glaucoma should not only be to preserve visual function by slowing or halting progression of the disease but should also include the maintenance or enhancement of QOL. Patients should be linked to vision rehabilitation and psychosocial support services. Demand based visual rehabilitation like hand held magnifiers for near work and hand held telescopes for distance magnification will enable patients to function independently and be a more integral part of society [26]. Patients with glare concerns can be provided with glare filters. Patients with advanced visual loss can be linked to blindness agencies for orientation and mobility training using cane, which is a valuable means of enabling patients to travel independently [27]. Appropriate psychosocial support for patients will also help them fight the anxiety brought by the fear of progressive losing of the remaining vision.

## Abbreviations

GQL-15: Glaucoma specific quality of life 15
HRQOL: Health-related quality of life
IOP: Intra ocular pressure
LOCS: Lens opacity classification system
MD: Mean deviation
VCDR: Vertical cup to disc ratio
VRQOL: Vision related quality of Life
WHO: World Health Organization
